# Predictors of Super-Responder Status to Anti-IL-23 Therapies in Moderate-to-Severe Plaque Psoriasis: A Real-World Monocenter Study

**DOI:** 10.3390/jcm14186371

**Published:** 2025-09-10

**Authors:** Sara Di Giulio, Costanza Falcidia, Giulio Foggi, Matteo Bianco, Luigi Gargiulo, Mario Valenti, Antonio Costanzo, Alessandra Narcisi, Luciano Ibba

**Affiliations:** 1 Dermatology Unit, IRCCS Humanitas Research Hospital, 20089 Rozzano, Italy; sara.digiulio@humanitas.it (S.D.G.); costanza.falcidia@humanitas.it (C.F.); giulio.foggi@humanitas.it (G.F.); matteo.bianco@humanitas.it (M.B.); mario.valenti@hunimed.eu (M.V.); antonio.costanzo@hunimed.eu (A.C.); alessandra.narcisi@hunimed.eu (A.N.); 2 Department of Biomedical Sciences, Humanitas University, 20072 Pieve Emanuele, Italy

**Keywords:** Anti-IL-23, biologics, guselkumab, psoriasis, risankizumab, super-responder, tildrakizumab

## Abstract

**Background/Objectives:** Psoriasis is a chronic immune-mediated skin disease with an estimated global prevalence of 3%. Real-world studies have demonstrated that biologic therapies have transformed the management of moderate-to-severe psoriasis by providing optimal disease control and a favorable safety profile. However, a new challenge lies in identifying those most likely to achieve an early and sustained response, defined as ‘super-responders’ (SRs). This is particularly relevant given recent evidence suggesting that IL-23 inhibitors may have long-term disease-modifying effects by acting on tissue-resident memory T cells. Identifying positive and negative baseline predictors associated with achieving SR status in patients treated with anti-IL-23 agents. **Methods:** This retrospective observational study analyzed data from the electronic medical records of IRCCS Humanitas Research Hospital between June 2021 and June 2025. A total of 611 patients with moderate-to-severe psoriasis who were treated with risankizumab, guselkumab or tildrakizumab were included in the study. Clinical assessments were conducted at baseline and weeks 16, 28 and 52. SR status was defined as achieving a PASI score of ≤1 at week 16, with this score being maintained through weeks 28 and 52. **Results:** Of the 611 enrolled patients, 390 (63.8 %) achieved SR status. In multivariate logistic regression, disease duration ≤ 2 years was the strongest independent predictor (Odds Ratio [OR] 2.47, *p* = 0.025), followed by bio-naïve status (OR 1.53, *p* = 0.019). Obesity (OR 0.71, 95 % CI 0.45–1.13) and cardiometabolic comorbidities (OR 0.93, 95 % CI 0.63–1.38) were not significantly associated with response after adjustments. No serious adverse events or treatment discontinuations occurred during 52 weeks of follow-up. **Conclusions:** Shorter disease duration (≤2 years) and bio-naïve status were identified as independent predictors of SR status. Identifying these patients could inform the development of personalized treatment strategies, including dose optimization and extended dosing intervals.

## 1. Introduction

Psoriasis is a widespread, chronic, immune-mediated skin disease. This condition has a global distribution, affecting over 60 million people worldwide with a considerable health and socioeconomic impact [1]. Men and women are equally affected, with a mean age of onset of 33 years [1]. Psoriasis is frequently associated with different comorbidities, including psoriatic arthritis (PsA), inflammatory bowel disease, psychiatric disorders and cardiometabolic diseases [2].

The pathophysiology of psoriasis involves an interaction between genetic and environmental factors, leading to the activation of immune mechanisms and cells that contribute to the development and maintenance of the disease [3,4]. Genome-wide association studies have identified over 80 genetic loci linked to psoriasis, accounting for 30% of disease heritability. Among these, the HLA-Cw06*02 allele represented the most significant risk factor, particularly for early-onset psoriasis [1,3].

The pathogenesis of the disease involves an interplay between the innate and adaptive immune systems, with T cells, dendritic cells, neutrophils, and keratinocytes playing central roles. This complex interaction between these cells is mediated by pro-inflammatory cytokines [4]. Among the cytokine networks implicated, the IL-23/IL-17 axis is particularly critical [5]. IL-23, composed of p19 and p40 subunits, maintains and expands populations of IL-17-producing cells (Th17, Tc17, ILC3). IL-17A form is abundantly expressed in psoriatic lesions and drives further inflammation by upregulating CCL20, a chemokine that recruits additional Th17 cells and amplifies the inflammatory response [1,5].

There are five recognized clinical subtypes of psoriasis, with plaque psoriasis (psoriasis vulgaris) being the most common, accounting for approximately 90% of cases [6]. It is immunologically dominated by the IL-23/IL-17 axis and clinically characterized by well-demarcated, erythematous plaques with silvery scaling, commonly involving the extensor surfaces of the lower and upper limbs, as well as the presacral regions, although any area can be affected [6].

The therapeutic management of psoriasis is primarily driven by disease severity, as defined by the “Rule of Tens” [7]. This classification system categorizes psoriasis as moderate-to-severe when there is involvement of more than 10% of the Body Surface Area (BSA), a Psoriasis Area and Severity Index (PASI) score greater than 10, or a Dermatology Life Quality Index (DLQI) score greater than 10 [7].

First-line treatment for moderate-to-severe psoriasis and cases associated with severe involvement of difficult-to-treat areas (such as the face/scalp, palms/soles, nails and genitalia) traditionally relied on conventional systemic therapies [8]. These included methotrexate, a folate antagonist; acitretin, an oral retinoid particularly effective in palmoplantar psoriasis and often combined with phototherapy; cyclosporine, a calcineurin inhibitor; apremilast, a phosphodiesterase-4 (PDE-4) inhibitor; and fumarates [2,8].

However, in the last twenty years, the management of this condition has been revolutionized by the introduction of biological therapies into the therapeutic landscape.

Biologics are considered first-line treatment for moderate-to-severe forms when conventional systemic therapies prove to be ineffective, contraindicated, or not tolerated [9]. Among innovative systemic treatments approved in Italy, biological agents targeting TNF-α, IL-17, IL-12/23 and IL-23 are currently available [10,11]. Deucravacitinib, a novel oral agent targeting Tyrosine Kinase (TYK) 2, is also a treatment option approved for the same indications as the drugs previously mentioned [10,11,12,13].

The IL-17 inhibitor drug class approved in Italy includes bimekizumab (which targets both IL-17A and IL-17F), brodalumab (which targets the IL-17 receptor), as well as ixekizumab and secukinumab (both targeting IL-17A) [11,14,15,16,17,18]. These agents act by inhibiting the signaling pathways of the IL-17 cytokine family and have been shown to induce a rapid and sustained reduction in PASI scores, while also demonstrating excellent efficacy on the articular manifestations of the disease. However, their use is associated with a slightly increased risk of mild adverse events (AEs), particularly fungal infections [19,20,21,22,23,24]. As for IL-23 inhibitors, the approved agents in Italy are guselkumab, risankizumab, and tildrakizumab; these drugs act by binding to the p19 subunit of IL-23, thereby inhibiting its interaction with the corresponding receptor [11,25,26,27,28]. Recent real-world studies have demonstrated the excellent effectiveness, safety, and long-term durability of guselkumab, tildrakizumab, and risankizumab, with comparable outcomes across agents [27,28,29,30,31,32]. Ustekinumab is an IL-23-targeting biologic that acts through a distinct mechanism by binding to the p40 subunit, which is shared by both IL-12 and IL-23. This interaction inhibits the binding of these cytokines to their respective receptors [33,34]. In terms of PASI score reduction, its efficacy is generally considered inferior to that of agents selectively targeting IL-23 [14,16,25,35].

To date, evidence from recent literature increasingly supports the superior effectiveness and safety profile of IL-17 and IL-23 inhibitors in comparison to conventional systemic therapies and anti-TNF agents for the management of psoriasis [15,16,17,18,19,25,26,27]. However, no current guidelines recommend favoring one innovative systemic biological agent over another based on patient characteristics or disease presentation, whether comparing drugs within the same class or between the two classes of IL-17 and IL-23 antagonists [9,10,11]. The only clear limitation is that IL-17 inhibitors should not be recommended in patients with a history of chronic inflammatory bowel diseases [9]. Nonetheless, according to recent real-world evidence, IL-23 inhibitors appear to be associated with a longer drug survival compared to IL-17 inhibitors [29]. This effect is linked to IL-23’s suppression of regulatory T (Treg) cell differentiation and its promotion of the differentiation, survival, and expansion of pathogenic Th17 and tissue-resident memory T (TRM) cells. These mechanisms contribute to the chronicity of autoimmune and inflammatory diseases [36]. Consequently, anti–IL-23 therapies, through their enhanced ability to modulate the balance between Treg and pathogenic TRM cells, achieve superior long-term maintenance of clinical response compared to IL-17A inhibition [36].

When assessing a patient’s response to psoriasis biological treatment, the rapidity of disease control and long-term durability of the therapeutic effect should be considered [37]. In this regard, some patients achieve almost complete control of cutaneous manifestations within the first months of biologic treatment, reaching PASI scores < 1; similarly, there are patients who, once disease control is achieved with a particular agent, experience no further flares for prolonged periods while remaining on the same therapy [37,38,39].

In particular, the sustained response over time is thought to be related to the action of anti-IL-23/IL-17 agents on skin-resident memory T cells. By blocking the pro-inflammatory effects of these cytokines, a long-term reprogramming of the patient’s immune system may be achieved. This immunological shift could potentially protect the individual from disease relapse not only during active treatment, but also after discontinuation of therapy [40,41]. Patients who meet the therapeutic goals of rapid disease control and/or sustained response have been recently defined in some studies as “super-responders” (SRs) [38,39,42,43,44]. These articles report associations between certain baseline patient characteristics and SR status during therapy. Positive associations were found with bio-naïve status, shorter disease duration (≤2 years), lower baseline PASI scores and HLA type. However, this evidence is limited, restricted to some agents in the class, and based on preliminary data [38,39,42,43,44].

The aim of this study is to systematically analyze a large cohort of patients treated with IL-23 inhibitors to determine whether there are factors, either intrinsic to the patient or extrinsic, such as biologic choice, that may facilitate the identification of SRs.

## 2. Materials and Methods

### 2.1. Patients and Study Design

This retrospective monocentric study was conducted by analyzing database records of adult patients with moderate-to-severe plaque psoriasis at IRCCS Humanitas Research Hospital between June 2021 and June 2025.

A total of 611 patients who had been treated with anti-IL-23 agents (guselkumab, risankizumab or tildrakizumab) for at least one year were enrolled in the study.

Eligibility for biological treatments was determined according to the Italian Guidelines for the management of moderate-to-severe plaque psoriasis [11].

Guselkumab, risankizumab and tildrakizumab were administered in accordance with their respective Summary of Product Characteristics [45,46,47].

Patient demographic characteristics at baseline, including age, sex, body mass index (BMI), and presence of concomitant PsA, were extracted from institutional electronic medical records. Additional baseline data included disease duration, PASI (Psoriasis Area and Severity Index) score and the involvement of at least one difficult-to-treat area (scalp/face, nails, genitalia, or palms/soles) [48]. Chronic infections and cardiometabolic comorbidities were also recorded, including arterial hypertension, obesity (BMI ≥ 30), type-2 diabetes mellitus, hypercholesterolemia and MAFLD (Metabolic-Associated Fatty Liver Disease).

### 2.2. Clinical Assessment of SR Status

Clinical assessments were conducted at baseline, weeks 16, 28, and 52. The primary endpoint was the identification of SR status, which was defined as achieving a PASI score of ≤1 by week 16 and maintaining this outcome at both weeks 28 and 52. This definition aligns with the response criteria proposed in the recent literature [49].

### 2.3. Safety Assessment

Safety was assessed by evaluating the incidence of adverse events (AEs), including serious AEs, AEs leading to discontinuation and laboratory abnormalities throughout the 52 weeks, as documented in medical records.

### 2.4. Statistical Analysis

All analyses were conducted in accordance with the intention-to-treat principle. Continuous variables were presented as mean and standard deviation (SD), while categorical variables were expressed as absolute numbers and percentages. Statistical comparisons of categorical data were performed using the chi-square and Exact Fisher’s tests. Continuous variables were analyzed with Student’s *t*-test or Mann–Whitney U test, depending on the distribution.

The correlation between the SR status and several variables including age, obesity (yes/no), cardiometabolic comorbidities (yes/no), concomitant PsA (yes/no), disease duration (≤2 years or >2 years), the presence of difficult-to-treat areas (yes/no), the previous exposure to other biological drugs (yes/no), PASI at baseline (≥12 or <12) and the current anti-IL-23 inhibitor (guselkumab, risankizumab or tildrakizumab) was assessed.

Variables with a *p*-value of less than 0.2 in the univariate analysis were included in a multivariate logistic regression model. Odds Ratios (ORs) and 95% Confidence Intervals (CIs) were reported.

A *p*-value < 0.05 was considered statistically significant. Data analysis was performed using Stata/SE 18.0, and tables and figures were generated using Microsoft Excel and GraphPad Prism 10.2.

### 2.5. Ethical Consideration

Institutional review board approval was exempted as the study protocol did not deviate from standard clinical practice. All patients received guselkumab, risankizumab or tildrakizumab as in good clinical practice, following European guidelines. All included patients had provided written consent for a retrospective study of data collected during routine clinical practice (demographics, clinical scores). The study was performed in accordance with the Helsinki Declaration of 1964 and its later amendments. Data collection and handling complied with applicable laws, regulations, and guidance regarding patient protection, including patient privacy.

## 3. Results

### 3.1. Study Population

A total of 611 adult patients with moderate-to-severe plaque psoriasis were included in the study. All patients were treated with an anti-IL-23 agent (risankizumab, guselkumab, or tildrakizumab) and completed at least 52 weeks of follow-up. Patients who were treated with anti-IL-12/IL-23 (ustekinumab) were excluded from enrolment. The most commonly prescribed drug among them was risankizumab (380 patients, 62.2%), followed by guselkumab (147 patients, 24.1%) and tildrakizumab (84 patients, 13.8%).

The majority of the study population were male (64%) with a mean age of 53.83 years (SD 15.17). The mean BMI was 26.94 kg/m^2^ (SD 5.66). Overall, almost 15% of patients had concomitant PsA, while 78.2% had at least one difficult-to-treat area involved at baseline. Slightly more than half of our cohort (51.4%) presented with at least one cardiometabolic comorbidity, including arterial hypertension, obesity (BMI ≥ 30), type 2 diabetes mellitus, hypercholesterolemia, or MAFLD.

Four hundred and twenty patients (68.7%) were bio-naïve to biologics at the start of anti-IL-23 therapy. The mean disease duration at baseline was 19.78 years (SD, 14.30), and the mean PASI score at baseline was 12.17 (SD, 6.78), indicating moderate-to-severe disease severity across the cohort.

Additional demographic and clinical features of the study population are shown in Table 1.

### 3.2. “Super-Responder” Status

Among the 611 patients treated with anti-IL-23 agents, a total of 390 (63.8%) met the criteria for SR status. This was defined as achieving an absolute PASI score of ≤1 at week 16, with this outcome being maintained at both weeks 28 and 52.

**Figure 1:**
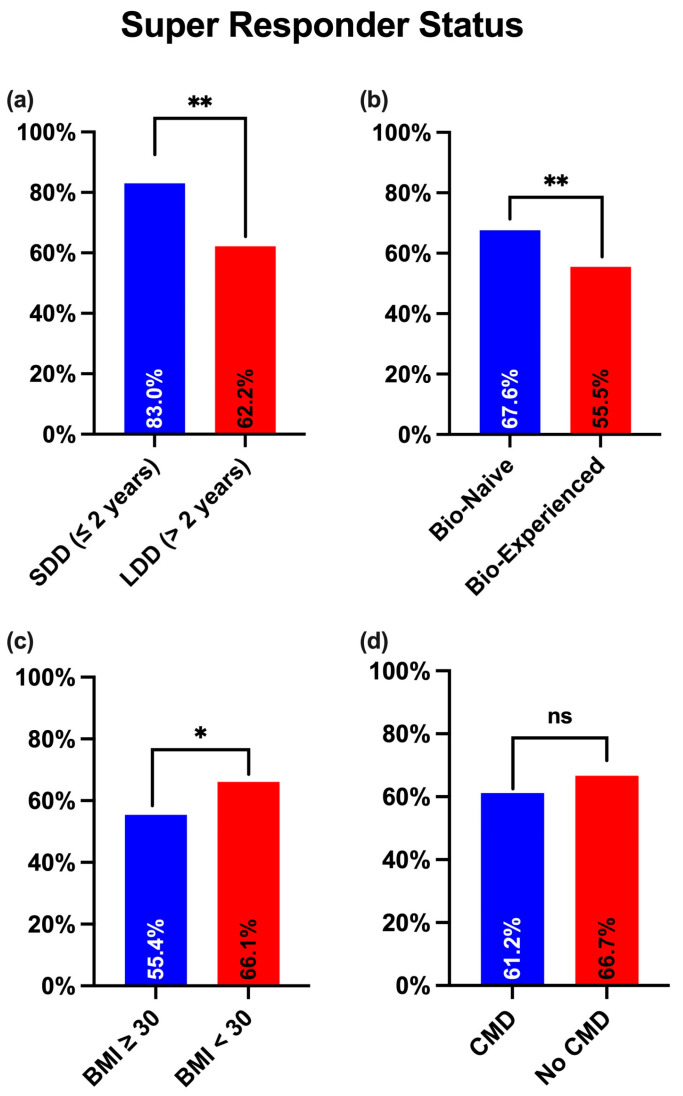
Proportion of patients achieving super-responder status according to disease duration (**a**), bio-naïve status (**b**), obesity (**c**) and the presence of cardiometabolic comorbidities (**d**). SDD: Short Disease Duration; LDD: Long Disease Duration; BMI: Body Mass Index; CMD: Cardiometabolic comorbidities; * *p* < 0.05; ** *p* < 0.01; ns: not significant.

Univariate analysis revealed that various baseline characteristics were associated with a significantly higher likelihood of achieving SR status. Patients with a disease duration of ≤2 years were significantly more likely to achieve the SR status than those with a disease duration of more than 2 years (83% vs. 62.2%, *p* = 0.004) (Figure 1a). Similarly, bio-naïve patients had higher SR rates than patients who had previously failed another biological treatment (67.6% vs. 55.5%, *p* = 0.004) (Figure 1b). Obesity was associated with a lower probability of achieving SR status (55.4% vs. 66.1% in non-obese patients, *p* = 0.024) (Figure 1c). Lastly, although the presence of cardiometabolic comorbidities did not reach statistical significance, a trend was observed (61.2% vs. 66.7% in patients without cardiometabolic comorbidities, *p* = 0.156) (Figure 1d).

Other baseline variables, including sex (male vs. female, *p* = 0.341), the presence of concomitant PsA (*p* = 0.466), baseline PASI severity (≥12 vs. <12, *p* = 0.774), the involvement of difficult-to-treat areas (*p* = 0.427) and the specific anti-IL-23 agent used (guselkumab, risankizumab or tildrakizumab, *p* = 0.781), were not significantly associated with SR status. Similarly, there was no significant difference in mean age at baseline between SRs and non-SRs (53.41 vs. 54.58 years, *p* = 0.359). Complete data for the univariate analysis are shown in Table 2.

**Figure 2:**
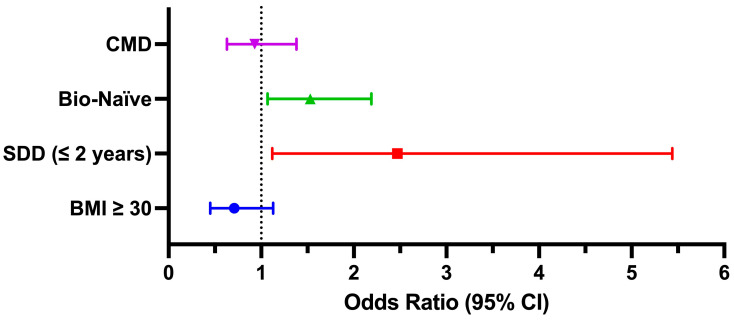
Forest plot showing odds ratios and 95% confidence intervals for baseline variables included in the multivariate logistic regression. Disease duration of ≤2 years and bio-naïve status were independently associated with a higher probability of achieving super-responder status. CMD: Cardiometabolic comorbidities; SDD: Short Disease Duration; BMI: Body Mass Index; CI: Confidence Interval.

Based on a *p*-value threshold of <0.2 in the univariate analysis, the following four independent variables were selected for the multivariate logistic regression model: disease duration (≤2 years vs. >2 years), bio-naïve status (yes/no), obesity (yes/no), and the presence of at least one cardiometabolic comorbidity (yes/no). In the multivariate model, shorter disease duration remained a strong independent predictor of SR status (OR: 2.47, 95% CI: 1.12–5.44, *p* = 0.025). Bio-naïve patients also had significantly higher odds of achieving this outcome (OR: 1.53, 95% CI: 1.07–2.19, *p* = 0.019). In contrast, the presence of cardiometabolic comorbidities (OR: 0.93, 95% CI: 0.63–1.38, *p* = 0.715) and obesity (OR: 0.71, 95% CI: 0.45–1.13, *p* = 0.148), while included in the model, were not independently associated with SR status in the adjusted model (Figure 2).

### 3.3. Safety

In terms of safety, no serious AEs or treatment discontinuations due to AEs were reported during the 52-week follow-up period in any of the 611 patients treated with anti-IL-23 agents (guselkumab, risankizumab, or tildrakizumab) (Table 3).

The overall incidence of AEs was low and comparable across the three treatment groups. The most frequently reported AE were upper respiratory tract infections (URTIs), occurring in 15 patients (2.5%), followed by headaches in 5 patients (0.8%). Regarding chronic infections, 35 patients (5.7%) tested positive for TB QuantiFERON at baseline. However, no cases of reactivation were reported during the one-year follow-up period, as confirmed by annual chest radiographs and pulmonology visits. Additionally, 54 patients (8.8%) had a history of hepatitis B virus infection, and 10 patients (1.6%) had a history of hepatitis C virus exposure. These patients underwent regular liver function tests and hepatology evaluations, and no evidence of viral reactivation was observed in any case throughout the treatment period.

## 4. Discussion

The psoriasis population displays significant heterogeneity in baseline demographic and clinical characteristics, including disease duration, BMI, prior exposure to biologics, and the presence of comorbidities [50]. These intrinsic factors can significantly influence both the rapidity and durability of individual treatment responses, as well as safety outcomes, resulting in variable disease control even among patients receiving the same biological therapy [50]. For this reason, identifying predictors of early and sustained treatment response among these variables is crucial to optimizing therapeutic strategies.

In recent years, a growing interest has emerged in identifying SR patients, to improve the management of biological therapies [49]. The rationale behind defining this subgroup is based on increasing evidence that early and targeted treatment can lead to more effective and durable disease control. However, this concept remains an evolving one without a standardized definition [49,50,51,52]. As a result, recent studies attempting to identify this patient class early have relied on heterogeneous effectiveness endpoints [49].

From the literature, it appears that the concept of SR is often defined without considering both the rapidity of onset and the durability of response over time. In particular, numerous studies have linked this definition principle to an early response, emphasizing the rapid onset of action of anti-IL-17 agents [53,54]. Mastorino et al. applied this concept to bio-naïve patients treated with both anti-IL-23 and anti-IL-17 biologics, who exhibited early improvement, defined as achieving a PASI 100 at week 16, which was maintained up to week 28. When analyzing only these initial, though unsustained, responses, they found that patients classified as SRs were more likely to be treated with anti-IL-17 agents [54]. Similarly, Rampoti et al. investigated clinical predictors of the initial response to brodalumab, defining SRs as patients who achieved an absolute PASI ≤1 after 12–16 weeks of treatment [53].

However, emerging molecular evidence shows that anti-IL-23 agents can modulate Th17 cell activity, contributing to long-term disease control and potentially enabling prolonged remission even after treatment discontinuation [55]. Although the exact mechanisms are not yet fully understood, recent research suggests that IL-23 inhibitors might have a higher potential for disease modification compared to anti-IL-17 agents [55].

Based on this evidence, we chose to focus our study exclusively on patients treated with anti-IL-23 agents, aiming to better characterize those who achieve both an early and sustained clinical response.

One of the earliest structured attempts to define the SR concept is represented by the GUIDE study, a Phase IIIb multicenter trial [38,39]. This study investigated the long-term disease control and potential disease-modifying effects of early guselkumab treatment. In its initial phase, patients received guselkumab 100 mg at weeks 0, 4, 12, and 20. Those who achieved a PASI score of 0 at both weeks 20 and 28 were classified as SRs [38,39]. Upon analysis of baseline demographic and clinical features, SRs were more frequently bio-naïve and had shorter disease duration compared to non-SRs [56].

In the second phase of the GUIDE study, patients who achieved SR status were randomized to receive guselkumab 100 mg either every 8 weeks or every 16 weeks, in order to evaluate whether disease control could be maintained with extended dosing intervals. The results revealed no significant difference in terms of PASI < 3 at week 68 between the two cohorts. This finding is likely attributable to the demonstrated reduction in CD8+ TRM cells in the skin of both groups; an effect believed to be driven by early treatment and key to achieving durable immunological control [57].

In the real-world setting, most studies aiming to define this new concept have based the classification of SRs on PASI outcomes. However, the choice of endpoint, whether absolute (e.g., PASI ≤ 1 or PASI = 0) or relative (e.g., PASI 90 or PASI 100), varies considerably across them. Furthermore, the duration over which these responses must be sustained is inconsistent, limiting comparability across real-world studies [42,43,50,51,52,53,54,57,58,59]. In our study, we aimed to identify both positive and negative baseline predictors associated with achieving SR status, defined as achieving an absolute PASI ≤ 1 by week 16 and maintaining it through weeks 28 and 52, incorporating both early and sustained response criteria.

Following the results of the GUIDE trial, most real-life studies on SRs available in the literature have focused on patients treated with guselkumab [43,59]. Marcelli et al. conducted a real-world study aimed at characterizing the SR profile in patients receiving guselkumab. In their analysis, SRs were defined as patients who achieved an absolute PASI = 0 after 20 weeks of therapy. Based on this definition, 62% of patients were classified as SRs and 38% as nSRs. Additionally, after 204 weeks of follow-up, PASI 100 was maintained in 86.8% of SRs compared with 62.8% of nSRs. They found that the strongest predictors of SR status were concomitant PsA at baseline and bio-naïve status, and not having received an IL-17 inhibitor as the last biologic treatment prior to guselkumab [59]. More recently, Mortato et al. defined SRs as patients treated with guselkumab who achieved PASI 100 by week 20. Their study identified obesity (BMI ≥ 30), bio-experienced status, and higher baseline PASI scores as negative predictive factors for SR classification [43]. Loft et al. took a broader approach to this concept by applying the idea of SRs to long-term treatment durability. They defined SRs as patients who were treated with their first biologic for at least five years without a PASI score > 3 between 6 months and 5 years of treatment. The study found that these patients had fewer comorbidities compared to others [58]. Additionally, Talamonti et al. observed an earlier and more sustained response to ustekinumab in patients who tested positive for HLA-C*06:02 than in those who tested negative. The former group also had a lower mean age and PASI score at baseline [60].

Our findings were consistent with the GUIDE study, showing that patients treated with anti-IL-23 agents can frequently reach SR status, regardless of the specific drug used (tildrakizumab, risankizumab, or guselkumab). Moreover, a shorter disease duration (≤2 years) and bio-naïve status were significantly associated with SR status in the multivariate logistic regression. In contrast, we found no significant correlation between this status and gender, BMI, PASI at baseline, presence of cardiometabolic comorbidities, involvement of difficult-to-treat areas, or the co-presence of PsA. These results are aligned with other real-world studies, which have found no differences in terms of effectiveness among patients with involvement of difficult-to-treat areas or those with cardiometabolic comorbidities treated with anti-IL-23 agents [30,32,61,62].

Regarding safety, our findings are consistent with those reported in both randomized clinical trials and real-world studies, showing no major safety concerns or relevant differences among patients treated with the various anti-IL-23 agents [63]. In our study, reported AEs were infrequent and generally mild. Notably, no cases of latent tuberculosis, hepatitis B, or hepatitis C reactivation occurred during the entire follow-up period, even among patients who were positive at baseline screening for QuantiFERON-TB, hepatitis B virus or hepatitis C virus. These results, aligned with real-world evidence, further support the favorable tolerability profile of IL-23 inhibitors [64].

This study has different limitations. First, its retrospective and monocentric design may introduce information or selection biases, which limit the generalizability of our findings. Second, without a consensus definition, our classification of SRs was guided by both clinical expertise and critical appraisal of emerging proposals. As a result, using different thresholds could influence the statistical significance of the identified predictors. Furthermore, our study did not investigate the potential correlation with key genetic loci, particularly the HLA-Cw6 allele. Therefore, further research is needed to establish whether HLA-Cw6 status could be a predictive factor. Finally, long-term outcomes should be evaluated through extended follow-up to confirm whether patients who achieved SR status at weeks 28 or 52 maintain their response over time.

## 5. Conclusions

In this large real-world cohort of patients with moderate-to-severe plaque psoriasis treated with IL-23 inhibitors, nearly two-thirds achieved SR status, defined as an absolute PASI ≤ 1 by week 16 and maintained through weeks 28 and 52. Shorter disease duration and bio-naïve status emerged as independent predictors of this outcome, while obesity and cardiometabolic comorbidities did not significantly affect the likelihood of achieving or maintaining SR status after adjustment for these factors. These findings support the early use of IL-23 inhibitors to maximize the probability of achieving rapid and durable disease control. Future multicenter, prospective studies with longer follow-up are needed to validate these predictors, explore underlying immunological mechanisms, and assess whether identifying SR patients can guide personalized treatment strategies, including dose-spacing approaches.

## Figures and Tables

**Table 1:** Demographic and clinical characteristics of our cohort of patients treated with anti-IL-23 inhibitors.

Total Patients	611
	**N (%)**
Male	391 (64)
PsA	91 (14.9)
At least one difficult-to-treat area	478 (78.2)
Cardiometabolic comorbidities	314 (51.4)
Bio-Naïve	420 (68.7)
*Anti-IL-23 drug*	
Risankizumab	380 (62.2)
Tildrakizumab	84 (13.8)
Guselkumab	147 (24.1)
	**Mean (SD)**
Age, years	53.83 (15.17)
BMI, kg/m^2^	26.94 (5.66)
Disease duration, years	19.78 (14.30)
PASI at baseline	12.17 (6.78)

PsA: Psoriatic Arthritis; SD: Standard Deviation; BMI: Body Mass Index; PASI: Psoriasis Area and Severity Index.

**Table 2:** Univariate analysis of baseline categorical and quantitative variables associated with super-responder status.

Categorical Variables		
	**Super-Responder Status**	***p*** **-Value**
SDD (≤2 years)	39/47 (83%)	**0.004**
LDD (>2 years)	351/564 (62.2%)	
Bio-Naïve	284/420 (67.6%)	**0.004**
Bio-Experienced	106/191 (55.5%)	
BMI ≥ 30	72/130 (55.4%)	**0.024**
BMI < 30	318/481 (66.1%)	
Tildrakizumab	51/84 (60.7%)	0.781
Risankizumab	243/380 (64%)	
Guselkumab	96/147 (65.3%)	
Male	255/391 (65.2%)	0.341
Female	135/220 (61.4%)	
PASI ≥ 12	200/316 (63.3%)	0.774
PASI < 12	190/295 (64.4%)	
CMD	192/314 (61.2%)	**0.156**
No CMD	198/297 (66.7%)	
Difficult areas	309/478 (64.6%)	0.427
No difficult areas	81/133 (60.9%)	
PsA	55/91 (60.4%)	0.466
No PsA	335/520 (64.4%)	
**Quantitative Variables**		
	**Mean age (SD)**	***p*** **-value**
Super-Responder	53.41 (15.60)	0.359
Non-Super-Responder	54.58 (14.38)	

SDD: Short Disease Duration; LDD: Long Disease Duration; BMI: Body Mass Index; CMD: Cardiometabolic Comorbidities; PsA: Psoriatic Arthritis; SD: Standard Deviation. Variables with a *p*-value < 0.2 were selected for multivariate logistic regression (highlighted in bold).

**Table 3:** Safety profile of guselkumab, risankizumab and tildrakizumab throughout the study period.

AEs	Guselkumab (*n* = 147)	Risankizumab (*n* = 380)	Tildrakizumab (*n* = 84)
Total	6 (4.1%)	15 (4%)	3 (3.6%)
URTIs	4 (2.7%)	9 (2.4%)	2 (2.4%)
Headache	1 (0.7%)	3 (0.8%)	1 (1.2%)
Diarrhea	1 (0.7%)	2 (0.5%)	0
Reaction at injection site	0	1 (0.3%)	0
Severe AEs	0	0	0
AEs leading to discontinuation	0	0	0

AE: Adverse Event; URTI: Upper Respiratory Tract Infection.

## Data Availability

Additional data supporting the findings of this manuscript are available on reasonable request to the corresponding author.

## Abbreviations

The following abbreviations are used in this manuscript:

PsA	Psoriatic Arthritis
TNF	Tumor Necrosis Factor
IFN	Interferon
IL	Interleukin
BSA	Body Surface Area
PASI	Psoriasis Area and Severity Index
DLQI	Dermatology Life Quality Index
PDE-4	Phosphodiesterase-4
AEs	Adverse Events
Treg	Regulatory T cells
TRM	Tissue-Resident Memory T cells
SR	Super-Responder
BMI	Body Mass Index
MAFLD	Metabolic-Associated Fatty Liver Disease
SD	Standard Deviation
OR	Odds Ratio
CI	Confidence Interval
URTIs	Upper Respiratory Tract Infections
TB	Tuberculosis
HBV	Hepatitis B Virus
HCV	Hepatitis C Virus
SDD	Short Disease Duration
LDD	Long Disease Duration
CMD	Cardiometabolic Comorbidities
